# First-principles based simulations of electronic transmission in ReS_2_/WSe_2_ and ReS_2_/MoSe_2_ type-II vdW heterointerfaces

**DOI:** 10.1038/s41598-021-02704-2

**Published:** 2021-12-06

**Authors:** Dipankar Saha, Saurabh Lodha

**Affiliations:** 1 Department of Electrical Engineering, Indian Institute of Technology Bombay, Mumbai, 400076 India; 2 Department of Electronics and Telecommunication Engineering, Indian Institute of Engineering Science and Technology Shibpur, Howrah, 711103 India

**Keywords:** Engineering, Nanoscience and technology

## Abstract

Electronic transmission in monolayer ReS$_{2}$ and ReS$_{2}$ based van der Waals (vdW) heterointerfaces are studied here. Since ReS$_{2}$/WSe$_{2}$ and ReS$_{2}$/MoSe$_{2}$ type-II vdW heterostructures are suitable for near infrared (NIR)/short-wave infrared (SWIR) photodetection, the role of interlayer coupling at the heterointerfaces is examined in this work. Besides, a detailed theoretical study is presented employing density functional theory (DFT) and nonequilibrium Green’s function (NEGF) combination to analyse the transmission spectra of the two-port devices with ReS$_{2}$/WSe$_{2}$ and ReS$_{2}$/MoSe$_{2}$ channels and compare the near-equilibrium conductance values. Single layer distorted 1T ReS$_{2}$ exhibits formation of parallel chains of ‘Re’-‘Re’ bonds, leading to in-plane anisotropy. Owing to this structural anisotropy, the charge carrier transport is very much orientation dependent in ReS$_{2}$. Therefore, this work is further extended to investigate the role of clusterized ‘Re’  atoms in electronic transmission.

## Introduction

In recent years significant progress in micro and nano photonics has been observed owing to the optoelectronic devices based on atomically thin two-dimensional (2D) layered materials and their vertical stacks^1–14^. Such 2D materials can be metallic (graphene) or, semiconducting (e.g., black phosphorus, transition metal dichalcogenides, etc.) or, a combination of both^15–19^. The vdW heterointerfaces formed with vertical stacking of 2D materials may exhibit compelling new properties which are significantly different from those of the participant materials^8,20,21^. Strong interlayer coupling and fast charge transfer across vdW interface are the key features which largely determine performance efficiency of the heterostructures^8,9,20,22,23^. In oder to design ultrafast NIR and SWIR photodetectors, a theoretical study exploring various possible combinations of group-6 and group-7 monolayer transition metal dichalcogenides (TMDs) is presented in^20^. The detection and visualization of NIR/SWIR are crucial to successful design and implementation of various biomedical devices, sensors, and cameras for surveillance^24,25^. Type-II heterointerfaces are necessary for those applications. As the conduction band minimum and valence band maximum reside in different layers, a type-II heterointerface can offer efficient e$^{-}$ and h$^{+}$ separation at the junction^20,26,27^. Among different combinations of type-II vdW heterostructures, it has been found that both ReS$_{2}$/WSe$_{2}$ and ReS$_{2}$/MoSe$_{2}$ emerge as suitable candidates with efficient generation, separation, and collection of charge carriers^20^. Howbeit, the effects of interlayer coupling on the electronic structures of ReS$_{2}$/WSe$_{2}$ and ReS$_{2}$/MoSe$_{2}$ were not captured in^20^. Moreover, to quantify the near-equilibrium conductance values of those heterointerfaces, a detailed analysis of electronic transmission through the ReS$_{2}$/WSe$_{2}$ and the ReS$_{2}$/MoSe$_{2}$ needs to be conducted.

Thus, in this work, considering band dispersions and electron difference density (EDD) calculations, first we try to investigate the role of interlayer coupling at the vdW heterojunctions. Next, utilizing NEGF along with DFT, we have shown the electronic transmission of two-port devices with the ReS$_{2}$/WSe$_{2}$ and the ReS$_{2}$/MoSe$_{2}$ channels and compared their conductance values at near-equilibrium.

Besides, we have also investigated the anisotropic carrier transmission of the group-7 constituent material, that is distorted 1T ReS$_{2}$. But for the group-6 TMDs (e.g., MoS$_{2}$, WS$_{2}$, MoSe$_{2}$, and WSe$_{2}$), because of the symmetric honeycomb structure of semiconducting 2H phase, such in-plane anisotropy is generally not expected^20,28^. Furthermore, it is worth mentioning that orientation dependent electro-thermal transport in 2D layered materials can be useful for the purpose of designing low power, ultrathin next generation electronic devices^28–33^. Among various 2D materials, in-plane anisotropy owing to structural transformation is seen in the 1T$^{\prime }$ phase of the MoS$_{2}$^28,34^. Strong anisotropic conductance, due to clusterization of ‘Mo’  atoms along the transport direction, is reported in^28^. A similar trend can be seen in distorted 1T ReS$_{2}$, where the formation of parallel chains of ‘Re’-‘Re’ bonds can lead to orientation dependent anisotropic transport^29^. As reported in^29^, distorted 1T ReS$_{2}$ exhibits direction dependent I-V as well as transfer characteristics. Such experimental observation motivated us to explore the electronic properties of single layer distorted 1T ReS$_{2}$ and investigate the role of clusterized ‘Re’  atoms in carrier transmission.

## Methodology

In order to coduct first-principles based DFT calculations, the software package “QuantumATK”  was used^35,36^. For all the unit cells and the supercells, computation of electronic structures and geometry optimizations were performed using the generalized gradient approximation (GGA) as exchange correlation along with the Perdew-Burke-Ernzerhof (PBE) functional^37^. Moreover, LCAO (linear combination of atomic orbitals) based numerical basis sets were utilized in this study to obtain results at the cost of reasonable computational load^28,35^. To attain good accuracy of quantum transport and electronic structure calculations, the OPENMX (Open source package for Material eXplorer) code was used as the norm-conserving pseudopotentials^38,39^. The basis sets for Mo, S, W, Se, and Re were taken as “s3p2d1”, “s2p2d1”, “s3p2d1”, “s2p2d1”, and “s3p2d1”  respectively. Besides, the density mesh cut-off value was set to 200 Hartree and the k-points in Monkhorst-Pack grid were set to 9$\times$9$\times$1 (X-Y-Z) for the unit cells and 9$\times$9$\times$3 for the heterostructures. Sufficient vacuum was incorporated along the direction normal to the in-plane for the purpose of avoiding spurious interaction between periodic images. Furthermore, to include the effects of vdW interactions among different monolayers, the Grimme’s dispersion correction (DFTD2) was employed^40^. The DFTD2 scheme can provide accurate results with medium to large interatomic distances, like other van der Waals density functionals^20,41^.

Considering the two-port devices, transmission spectra along the channels were computed utilizing NEGF along with DFT. The k-points in Monkhorst-Pack grid were adopted as 1$\times$9$\times$150 for the device calculations. Apart from that, in order to solve the Poisson’s equation, Dirichlet boundary condition in the transport direction (Z direction) and periodic boundary conditions in the other two directions (X–Y directions) were assigned. In the framework of NEGF, taking into account the broadening matrices $\Gamma _{l,r}(E)=i[\Sigma _{l,r}-\Sigma _{l,r}^{\dagger }]$ for the left and the right electrodes (set by computing the self energy matrices $\Sigma _{l}$ and $\Sigma _{r}$)^42,43^, the electronic transmission can be calculated as,

1 $$\begin{aligned} T_{e}(E)=\text {Tr}[\Gamma _{l}(E)\, G(E)\,\Gamma _{r}(E)\, G^{\dagger }(E)]\;, \end{aligned}$$

where *G*(*E*) and $G^{\dagger }(E)$ denote retarded Green’s function and advanced Green’s function respectively^28,42,44^. Moreover, utilizing the concept of linear coherent transport, the conductance due to charge carriers and the Seebeck coefficient can be calculated as^36,42,43^,

2 $$\begin{aligned} G_{e}(\mu )=q^{2}\times L_{f,0}, \end{aligned}$$

and

3 $$\begin{aligned} S=\frac{1}{qT}\times \frac{L_{f,1}}{L_{f,0}}. \end{aligned}$$

Considering the Fermi distribution function f($\mu$, E), the following expression can be used to determine $L_{f,0}$ and $L_{f,1}$^42,43^.

4 $$\begin{aligned} L_{f,n}(\mu )=\frac{2}{h}\times \int _{-\infty }^{+\infty }T_{e}(E)\;(E-\mu )^{n}\;\bigg (\frac{-\partial f(\mu ,E)}{\partial E}\bigg )dE \end{aligned}$$

## Results and discussion

**Figure 1:**
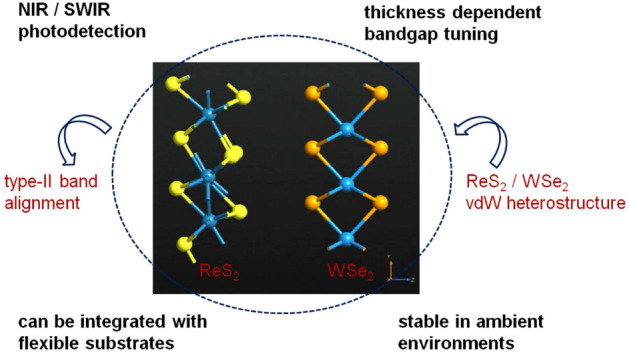
Vertical stacking of group-6 and group-7 monolayer TMDs to form a type-II vdW heterointerface (viz. ReS$_{2}$/WSe$_{2}$).

Figure 1 illustrates a typical type-II vdW heterostructure formed with vertical stacking of group-6 and group-7 monolayer TMDs, as well as highlights its salient features^20^. Considering NIR/SWIR photodetection, near-direct bandgap, larger band offset values, and feasibility of integrating with flexible substrates make monolayer ReS$_{2}$ (group-7 TMD)/ monolayer WSe$_{2}$ (group-6 TMD) heterointerface a potentially promising candidate for the next generation ultrathin optoelectronic device^2,9,17,20^. Even with the increase in number of layers of ReS$_{2}$ and WSe$_{2}$, we find that the near-direct type-II band alignment remains unaltered, hence ensuring efficient optical generation across the vdW interface^22^. Apart from that, monolayer ReS$_{2}$ (group-7 TMD)/ monolayer MoSe$_{2}$ (group-6 TMD) heterostructure can also be suitable for NIR/SWIR photodetection owing to its near-direct bandgap and superior optical absorption of the constituent MoSe$_{2}$ layer^20^.

**Figure 2:**
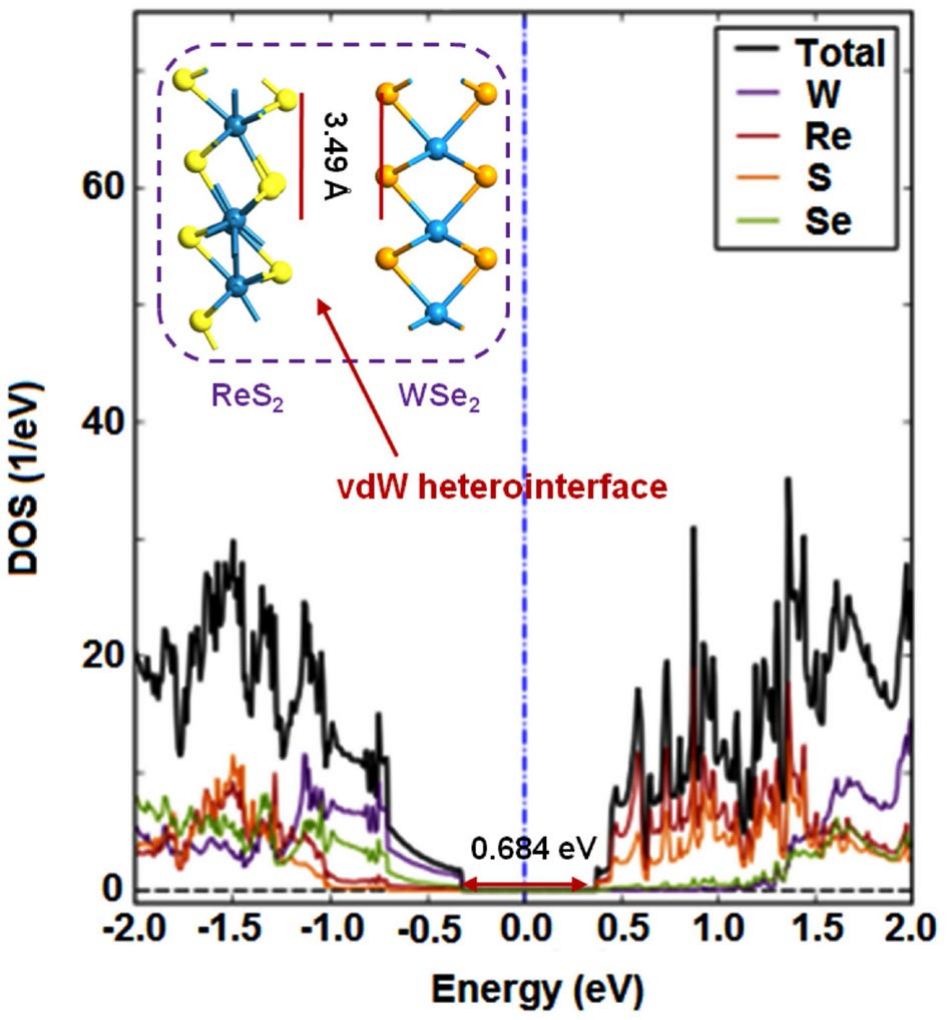
Projected DOS plots for ReS$_{2}$/WSe$_{2}$ vdW heterointerface.

**Figure 3:**
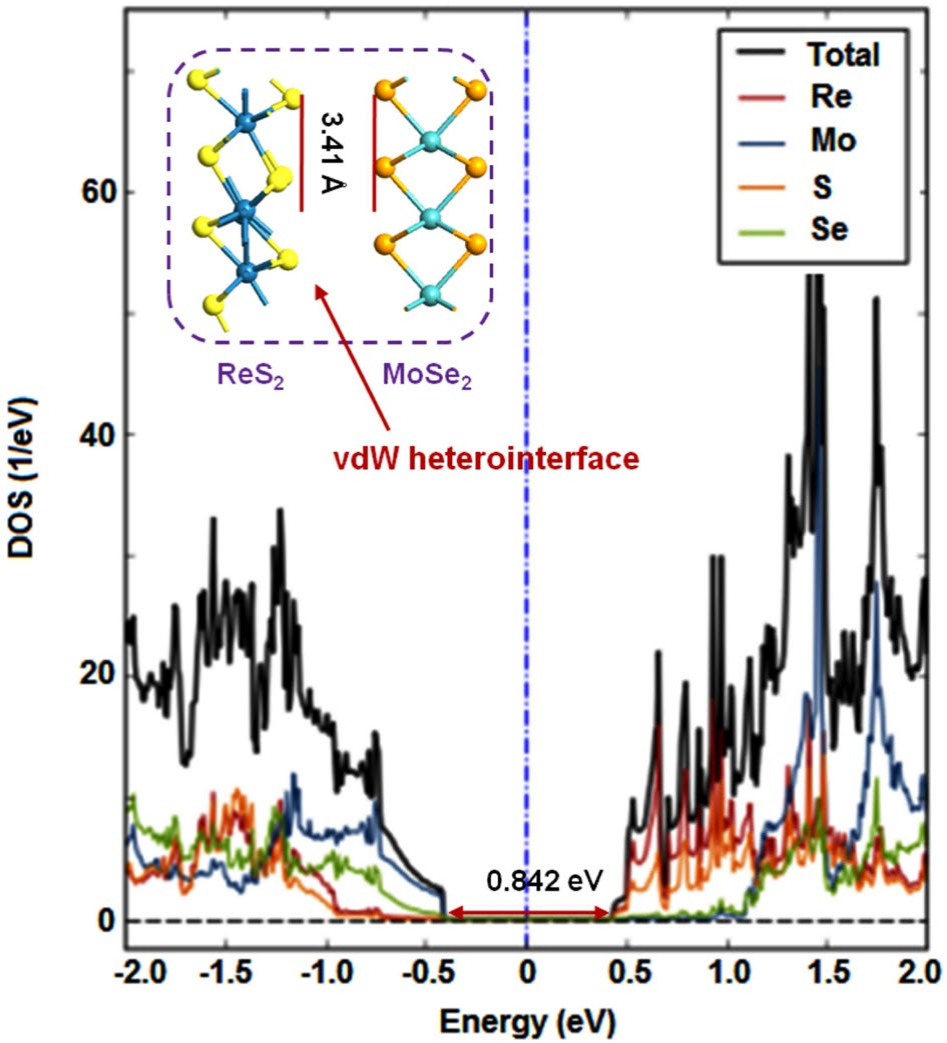
Projected DOS plots for ReS$_{2}$/MoSe$_{2}$ vdW heterointerface.

Figures 2 and 3 depict the electronic structures of ReS$_{2}$/WSe$_{2}$ and ReS$_{2}$/MoSe$_{2}$ considering element-wise contributions^45–47^. Both the projected density of states (DOS) plots exhibit type-II band alignments with energy gap values of 0.684 eV and 0.842 eV for the ReS$_{2}$/WSe$_{2}$ and the ReS$_{2}$/MoSe$_{2}$ heterostructures.

Besides, the in-plane lattice constants and the equilibrium interlayer distance for the hexagonal vdW heterointerface of ReS$_{2}$/WSe$_{2}$ (Fig.2 inset) are a = b = 6.600 Å  and 3.49 Å  respectively^20^. Those values for ReS$_{2}$/MoSe$_{2}$ interface (Fig. 3 inset) are a = b = 6.597 Å  and 3.41 Å^20^.

In order to determine the effect of interlayer coupling, next we carry out a detailed analysis which emphasizes mainly on the band dispersions and the electron difference density (EDD) calculations of the heterointerfaces^48^. We consider the equilibrium interlayer distances of ReS$_{2}$/WSe$_{2}$ and ReS$_{2}$/MoSe$_{2}$ as the references. Those equilibrium distances along with the in-plane lattice constants are computed while optimizing the geometries utilizing LBFGS (Limited-memory Broyden Fletcher Goldfarb Shanno) algorithm with force tolerance of 0.01 eV/Å  and stress tolerance of 0.001 eV/Å^3^. We also maintain the stacking patterns of the fully relaxed heterostructures same as those obtained form geometry optimization calculations. We then vary the interlayer distances (d_int_), and compute band dispersions of the vdW heterostructures (Fig. S1a and S1b). The changes in indirect and direct bandgap (I_gap_ and D_gap_) values, as we deviate from the equilibrium interlayer distances, are listed in Table 1.

I_gap_ and D_gap_ for ReS$_{2}$/WSe$_{2}$ are 0.68 eV and 0.83 eV, when the d_int_ is 3.49 Å  (equilibrium interlayer distance). On the other hand, I_gap_ and D_gap_ for ReS$_{2}$/MoSe$_{2}$ are 0.84 eV and 0.98 eV, when the d_int_ is 3.41 Å  (equilibrium interlayer distance). Now, as we shift the d_int_ around the equilibrium distances, we find that there are no noteworthy changes in band dispersions of ReS$_{2}$/WSe$_{2}$ and ReS$_{2}$/MoSe$_{2}$ (Fig. S1a and S1b), though the I_gap_ and the D_gap_ values do vary. We denote these changes as $|$ $\Delta$E_I_| and $|$ $\Delta$E_D_| (Table 1). Moreover, it is important to note that the variations in $|$ $\Delta$E_I_| and $|$ $\Delta$E_D_| are similar for the aforementioned type-II heterostructures.

**Figure 4:**
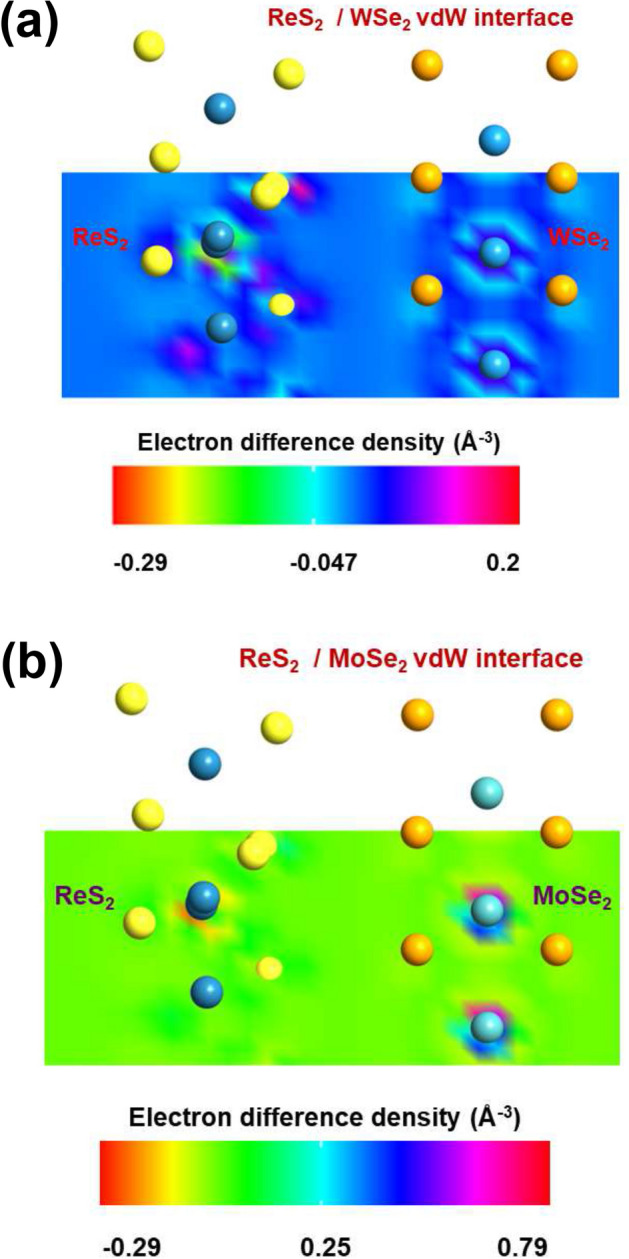
EDD plots showing charge re-distribution between the constituent layers of (**a**) ReS$_{2}$/WSe$_{2}$ and (**b**) ReS$_{2}$/MoSe$_{2}$.

Hence, we look into another important aspect, that is the EDD or the charge re-distribution between the constituent layers. As depicted in the cut plane diagrams of Fig. 4, negative and positive values of EDD indicate charge depletion and charge accumulation respectively. The EDD plot for ReS$_{2}$/WSe$_{2}$ (Fig. 4a) delineates minimum and maximum values of difference density as -0.29 and 0.2 Å$^{-3}$. Thus the corresponding average value is $\sim$ -0.04 Å$^{-3}$. On the other hand, EDD plot for ReS$_{2}$/MoSe$_{2}$ (Fig. 4b) shows minimum and maximum values of difference density as -0.29 and 0.79 Å$^{-3}$, with the average of $\sim$ 0.25 Å$^{-3}$. The larger average value of EDD for the ReS$_{2}$/MoSe$_{2}$ interface is essentially interpreting more charge distribution. Therefore, we can expect much stronger effect of interlayer coupling at the heterointerface of ReS$_{2}$/MoSe$_{2}$. Apart from that, the binding energy value of -0.403 eV ensures better energetic stability of ReS$_{2}$/MoSe$_{2}$ compared to that of ReS$_{2}$/WSe$_{2}$ (− 0.310 eV)^20^. In the following, we have further compared the electronic transmission in both the vdW heterointerfaces and observed a similar trend. At steady state, considering the two-port devices, the electron density in channel can be calculated as n_ch_ (r) = n_left_ (r) + n_b_ (r) + n_right_ (r), where n_left_ (r), n_right_ (r), and n_b_ (r) are the contributions from the extended left electrode, the extended right electrode, and the bound states^36^. The electrostatic potential as well as electron density of the central region will depend on occupation of bound states^36^. Therefore, strong interlayer coupling via charge re-distribution at the heterointerface can play significant role in determining the charge carrier transport^27,36^.

**Table 1:** Change in bandgap with the varying interlayer distances.

Interface	d_int_ (Å)	I_gap_ (eV)	D_gap_ (eV)	$|\Delta$E_I_| (eV)	$|\Delta$E_D_| (eV)
ReS$_{2}$/WSe$_{2}$	2.69	0.80	0.89	0.12	0.06
	3.49	0.68	0.83	0.00	0.00
	4.30	0.66	0.83	0.02	0.00
	5.10	0.66	0.82	0.02	0.01
ReS$_{2}$/MoSe$_{2}$	2.71	0.92	0.96	0.08	0.02
	3.41	0.84	0.98	0.00	0.00
	4.48	0.82	0.98	0.02	0.00
	5.18	0.81	0.97	0.03	0.01

**Figure 5:**
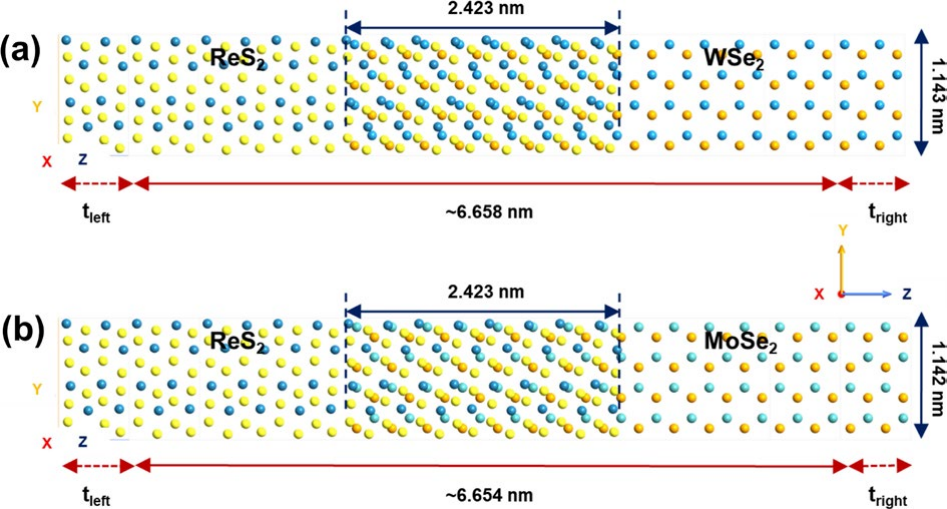
Atomistic models of the two-port devices with (**a**) ReS$_{2}$/WSe$_{2}$ and (**b**) ReS$_{2}$/MoSe$_{2}$ channels.

Next, to model the two-port devices, we take into account the geometry optimized vertical stacks of ReS$_{2}$/WSe$_{2}$ and ReS$_{2}$/MoSe$_{2}$ as illustrated in Figs. 2 and 3 (insets). As shown in Fig. 5, the group-7 and the group-6 TMD layers are extended to form the left and the right electrodes respectively. For the ReS$_{2}$/WSe$_{2}$ device, t_left_ (length of the left electrode) = t_right_ (length of the right electrode) is $\sim$ 6.60 Å. For the ReS$_{2}$/MoSe$_{2}$ device, that value is $\sim$ 6.59 Å. The length and the width of the channel region of ReS$_{2}$/WSe$_{2}$ two-port device are 6.658 nm and 1.143 nm (Fig. 5a). Considering the channel region of ReS$_{2}$/MoSe$_{2}$ two-port device, those values are 6.654 nm and 1.142 nm respectively (Fig. 5b). Besides, for those composite two-port device structures, the overlapping regions are maintained as $\sim$ 2.423 nm. It is worth mentioning that the mean absolute strain values on both the monolayer surfaces are 0.61% and 0.59% for ReS$_{2}$/WSe$_{2}$ and ReS$_{2}$/MoSe$_{2}$ channels^20^.

**Figure 6:**
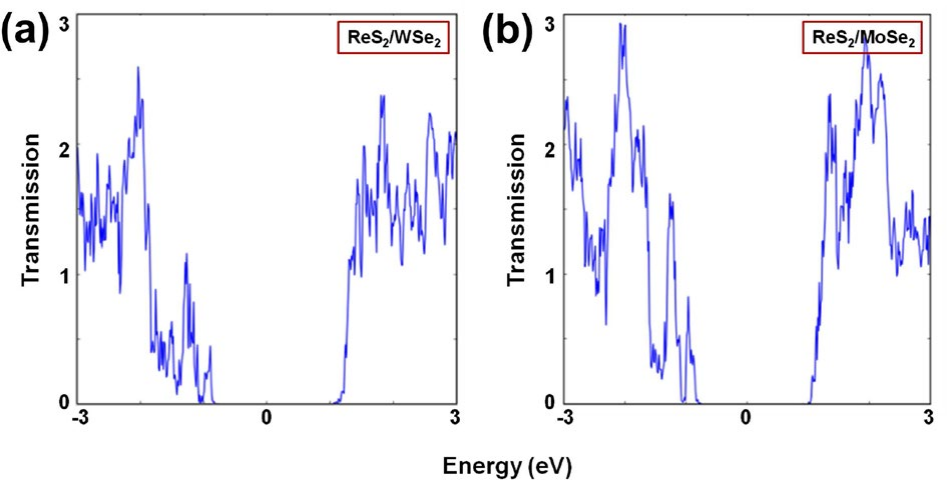
Transmission spectra of the two-port devices with (**a**) ReS$_{2}$/WSe$_{2}$ and (**b**) ReS$_{2}$/MoSe$_{2}$ channels.

Figure 6 shows the transmission spectra (zero bias) of the two-port devices, where we can observe that the transmission through ReS$_{2}$/MoSe$_{2}$ channel is slightly better than that in ReS$_{2}$/WSe$_{2}$ (cosidering a small energy range near the energy zero level). In order to quantify this we further calculate the near-equilibrium conductance and Seebeck coefficient values, as listed in Table 2. As discussed previously, S and G_e_ denote the Seebeck coefficient and the electrical conductance, computed utilizing the linear response approximation ($\sim$ 300 K)^28,43^. $\Delta$E_F_ represents the shift in energy level from the energy zero. Moreover, the plots of Seebeck coefficients for the energy range of $-3$–3 eV are shown in Fig. S2. Similar to the trends of transmission spectra (Fig. 6), the G_e_ values listed Table 2 reinforce that the ReS$_{2}$/MoSe$_{2}$ channel is more conducive to the charge carrier transport.

**Table 2:** Details of near-equilibrium conductance values.

Two-port devices	$\Delta$E_F_ (eV)	G_e_ (S)	S (V/K)
ReS$_{2}$/WSe$_{2}$ (length = 6.658 nm width = 1.143 nm)	0.9	2.906 $\, \times \,$ 10^–9^	– 0.0004686
	1.0	9.022 $\, \times \,$ 10^–8^	– 0.0002988
	1.1	1.023 $\, \times \,$ 10^–6^	– 0.0001835
	1.2	8.563 $\, \times \,$ 10^–6^	– 0.0001511
	1.3	3.540 $\, \times \,$ 10^–5^	– 4.829$\, \times \,$10^–5^
	1.4	5.180 $\, \times \,$ 10^–5^	– 1.856$\, \times \,$10^–5^
	1.8	7.897 $\, \times \,$ 10^–5^	– 9.423$\, \times \,$10^–6^
ReS$_{2}$/MoSe$_{2}$ (length = 6.654 nm width = 1.142 nm)	0.9	4.925 $\, \times \,$ 10^–8^	– 0.0004324
	1.0	1.396 $\, \times \,$ 10^–6^	– 0.0002594
	1.1	1.252 $\, \times \,$ 10^-5^	– 0.000103
	1.2	3.497 $\, \times \,$ 10^-5^	– 5.931$\, \times \,$10^–5^
	1.3	6.848 $\, \times \,$ 10^-5^	– 2.906$\, \times \,$10^–5^
	1.4	7.434 $\, \times \,$ 10^-5^	– 1.313$\, \times \,$10^–5^
	1.8	8.396 $\, \times \,$ 10^-5^	– 1.164$\, \times \,$10^–5^

Next, we have extended this theoretical study to demonstrate in-plane anisotropy and orientation dependent carrier transmission in single layer distorted 1T ReS$_{2}$. For the purpose of modeling the two-port devices with distorted 1T ReS$_{2}$ channels, we consider three different supercells viz. ‘ReS$_{2}$ supercell1’ (where the diamond-shaped DS ‘Re’ chains are perpendicular to the transport direction), ‘ReS$_{2}$ supercell2’  (where the DS ‘Re’ chains are parallel to the transport direction), and ‘ReS$_{2}$ supercell3’  (where the structure is obtained from the geometry optimized ReS$_{2}$/WSe$_{2}$ and the angle between a$_{1}$-b$_{1}$ and b$_{1}$-c$_{1}$ is $\sim$ 123$^\circ$)^20,29^.

**Figure 7:**
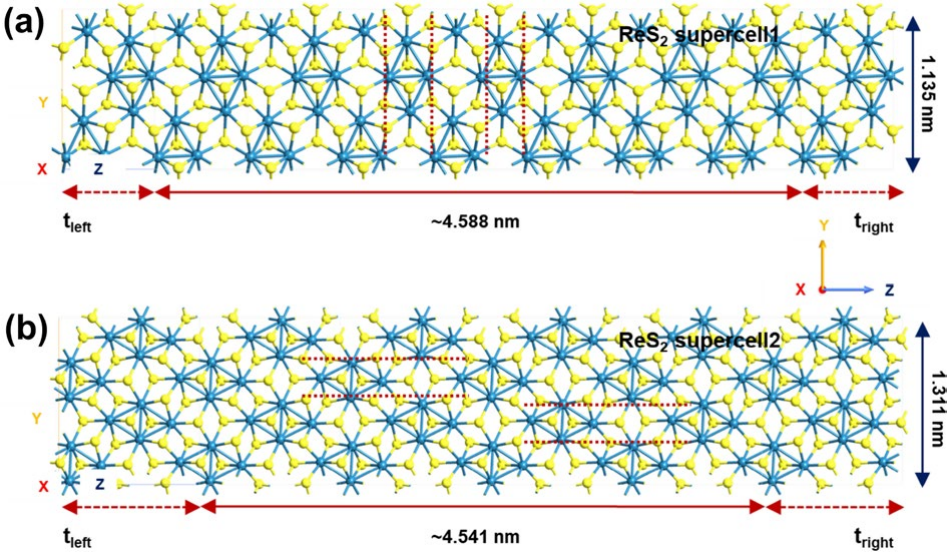
Atomistic models of (**a**) ReS$_{2}$ supercell1 and (**b**) ReS$_{2}$ supercell2, obtained by keeping the ‘Re’-‘Re’ bonds are either perpendicular or parallel to the transport direction.

**Figure 8:**
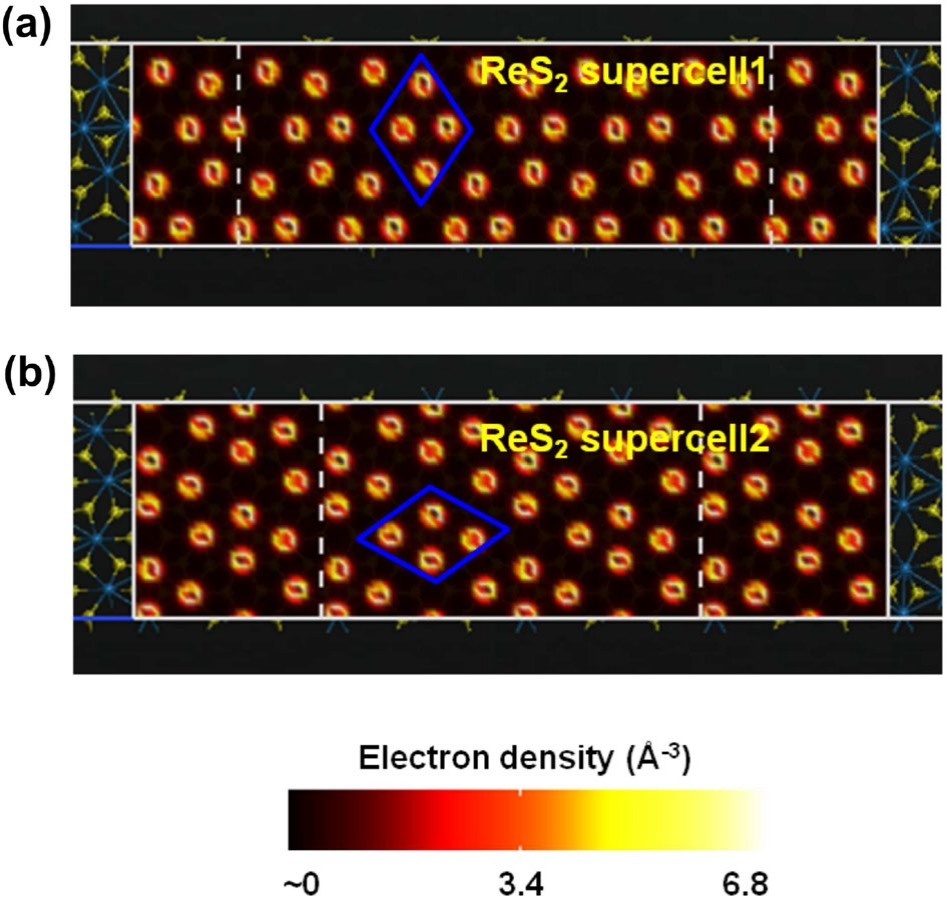
Electron density plots depicting the distribution of valence electrons around ‘Re’ atoms of ReS$_{2}$ supercell1 and ReS$_{2}$ supercell2.

**Figure 9:**
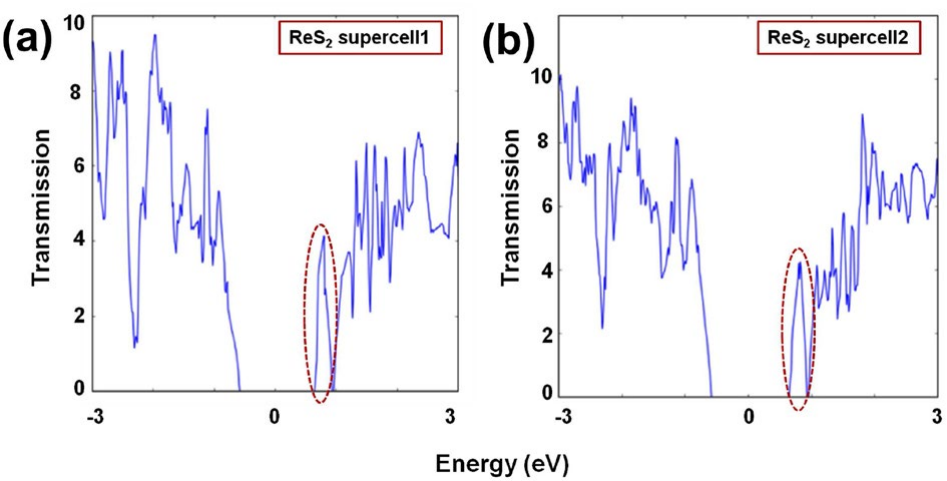
Transmission spectra (zero bias) of the two-port devices with (**a**) ReS$_{2}$ supercell1 and (**b**) ReS$_{2}$ supercell2 channel regions.

**Figure 10:**
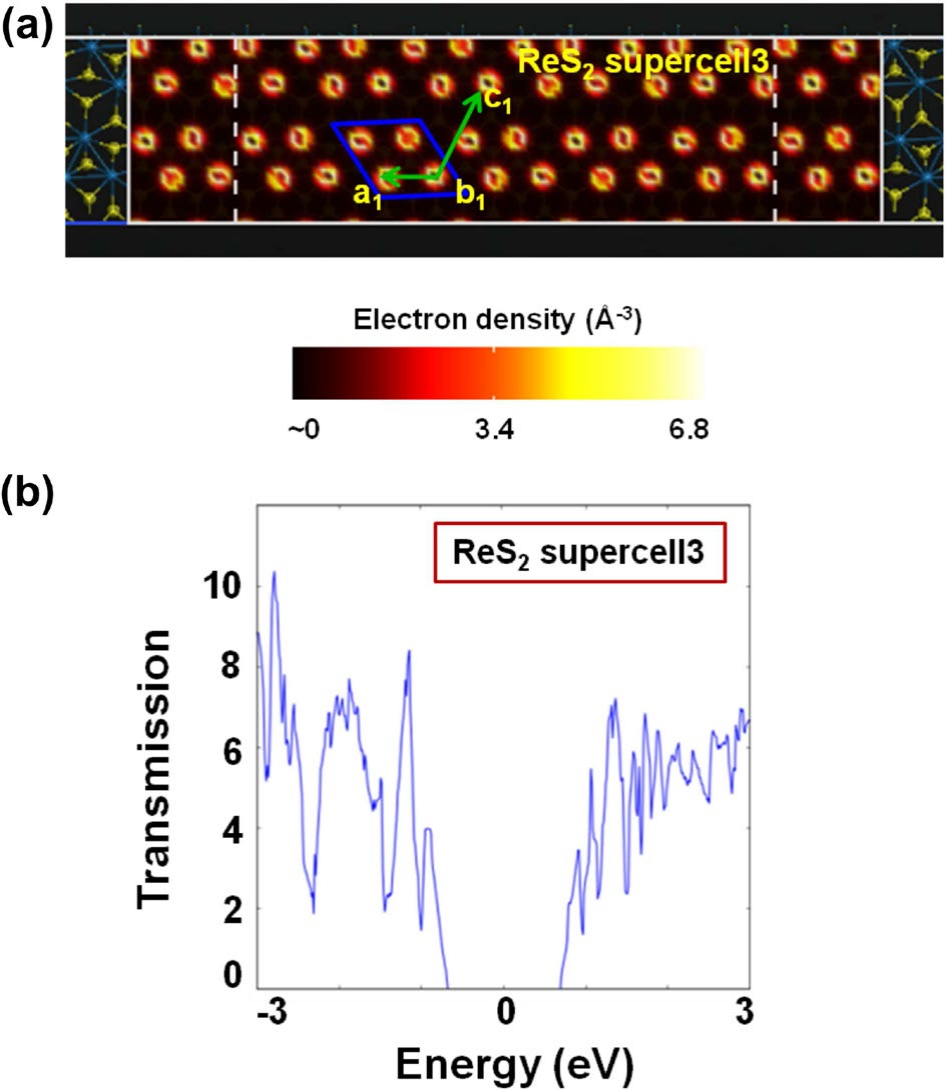
Plots illustrating the (**a**) electron density distribution around ‘Re’ atoms of ReS$_{2}$ supercell3 and (**b**) zero bias electronic transmission in two-port devices with ReS$_{2}$ supercell3 channel.

Figure 7 shows the atomistic models of the two-port devices with ‘ReS$_{2}$ supercell1’  (length = 4.588 nm and width = 1.135 nm) and ‘ReS$_{2}$ supercell2’  (length = 4.541 nm and width = 1.311 nm) channel regions. Figures 8 and 9 illustrate the electron density plots and zero bias transmission spectra of ‘ReS$_{2}$ supercell1’  and ‘ReS$_{2}$ supercell2’ . It is important to realize here that we have purposefully designed those supercell structures where the ‘Re’-‘Re’ bonds are either parallel or perpendicular to the transport direction (Z direction). However, for single layer distorted 1T ReS$_{2}$, intrinsically the DS ‘Re’ chains form a certain angle with the [100]/[010] axis^20,29^. Thus, as illustrated in Fig. 10a, ReS$_{2}$ supercell3’ (channel length = 4.620 nm and width = 1.143 nm) essentially exhibits the natural orientation of DS ‘Re’ chains.

Electron density plots of Fig. 8 depict how the valence electrons around ‘Re’ atoms are distributed across the channel regions. Apart from that, significant reduction in energy gap values can be observed for ‘ReS$_{2}$ supercell1’  and ‘ReS$_{2}$ supercell2’ (Fig. 9), owing to the large transmission states within the gap. Considering the transmission spectra as shown in Fig. 9, distinguishable transmission states within the range of 0.636 eV and 0.920 eV for ‘ReS$_{2}$ supercell1’  and 0.637 eV and 0.922 eV for ‘ReS$_{2}$ supercell2’, lower the energy gap values ($\sim$ 1.23 eV) of both the supercells.

However, the calculated energy gap for ‘ReS$_{2}$ supercell3’  (Fig. 10b) is $\sim$ 1.37 eV. More importantly, this value is similar to the bandgap of distorted 1T ReS$_{2}$ unit cell^20^. It further reinforces that the ‘ReS$_{2}$ supercell3’ has the geometry where orientation of DS ‘Re’ chains as well as distribution of valence electrons around ‘Re’ atoms (Fig. 10a), reflects that of the natural single layer.

## Conclusion

In this work, electronic transmission in single layer distorted 1T ReS$_{2}$ and distorted 1T ReS$_{2}$ based type-II vdW heterointerfaces are studied. We have demonstrated that the ReS$_{2}$/MoSe$_{2}$ heterostructure exhibits stronger effect of interlayer coupling at the heterointerface, owing to the larger average value of charge re-distribution between the constituent layers. Moreover, we have compared the electronic transmission through ReS$_{2}$/WSe$_{2}$ and ReS$_{2}$/MoSe$_{2}$ channels, and computed their near-equilibrium conductance values. We have found that the ReS$_{2}$/MoSe$_{2}$ channel is more conducive to the charge carrier transport. Apart from that, we have explored the in-plane anisotropy of the group-7 constituent material, that is the single layer distorted 1T ReS$_{2}$ and investigated the role of clusterized ‘Re’  atoms in electronic transmission. This study may further be extended for fewlayer ReS$_{2}$/WSe$_{2}$ and ReS$_{2}$/MoSe$_{2}$ channels, to explore their electronic properties and compare the near-equilibrium conductance values.

## Supplementary Information

Supplementary Information.

## Data Availability

All relevant data that support the findings of this study are available from the corresponding author upon request.
