# Supplementary material for: First-principles based simulations of electronic transmission in ReS2/WSe2 and ReS2/MoSe2 type-II vdW heterointerfaces
Source: Sci Rep. 2021 Dec 6;11:23455. doi: 10.1038/s41598-021-02704-2 (PMC8648936; doi:10.1038/s41598-021-02704-2)
Supplement: Supplementary file 1 — Supplementary Information. [file 41598_2021_2704_MOESM1_ESM.pdf]

Supporting Information

First-principles based simulations of electronic transmission in ReS_2/WSe_2 and $ReS_2/MoSe_2$ type-II vdW heterointerfaces

Dipankar Saha[1,2*] and Saurabh Lodha[1]

[1]Department of Electrical Engineering, Indian Institute of Technology Bombay, Mumbai-400076, India.

[2]Department of Electronics and Telecommunication Engineering, Indian Institute of Engineering Science and Technology Shibpur, Howrah-711103, India.

[*]Corresponding author (email: dipsah_etc@yahoo.co.in)

Band dispersions of the ReS_2/WSe_2 and ReS_2/$MoSe_2$ vdW heterointerfaces

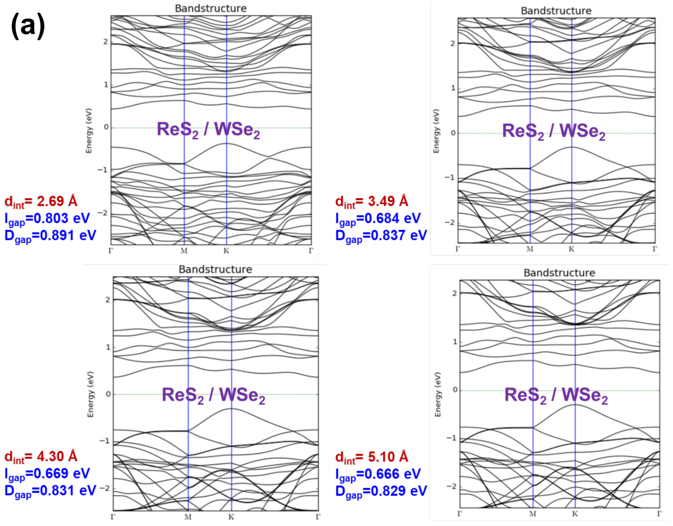

Figure S1 (a) : Band dispersions of the ReS_2/WSe_2 heterostructure with the varying interlayer distances.

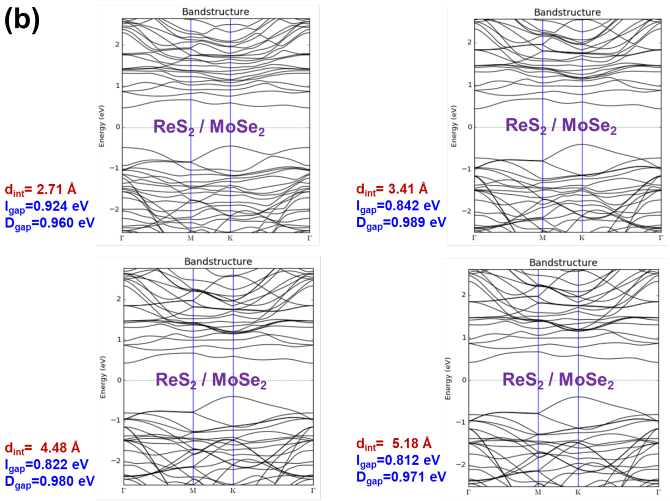

Figure S1 (b) : Band dispersions of the $ReS_2/MoSe_2$ heterostructure with the varying interlayer distances.

Seebeck coefficients plots (energy range of -3 eV to 3 eV)

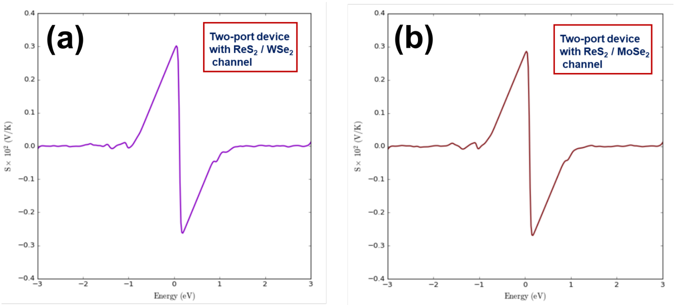

Figure S2 : Seebeck coefficient plots of the two-port devices with (a) ReS_2/WSe_2 and (b) $ReS_2/MoSe_2$ channels.
